# A systematic review and meta-analysis of *Toxoplasma gondii* infection among the Mexican population

**DOI:** 10.1186/1756-3305-5-271

**Published:** 2012-11-26

**Authors:** Ma de la Luz Galvan-Ramirez, Rogelio Troyo, Sonia Roman, Carlos Calvillo-Sanchez, Rosamaria Bernal-Redondo

**Affiliations:** 1 Department of Physiology, Neurophysiology Laboratory, Health Sciences University Center, University of Guadalajara, Sierra Mojada # 950 Edificio N, Col. Independencia, Guadalajara, Jalisco 44320, México; 2 “Federico Gómez” Children’s Hospital of Mexico, Mexico City, Mexico; 3 Department of Molecular Biology in Medicine, Civil Hospital of Guadalajara “Fray Antonio Alcalde”, University of Guadalajara, Hospital #278, Guadalajara, Jalisco 44280, Mexico

**Keywords:** Toxoplasmosis, *Toxoplasma* infection, Mexican population, Epidemiology, Meta-analysis

## Abstract

**Background:**

Toxoplasmosis is a disease caused by *Toxoplasma gondii* and at least one-third of the world’s population has detectable *T. gondii* antibodies. The seroprevalence of *T.gondii* ranges from 15% to 50% among the Mexican general population. The aim of this work was to determine the mean prevalence and weighted mean prevalence of *T. gondii* infection, and to evaluate the epidemiological transition of infection in Mexico.

**Methods:**

Pub Med, Lilacs, Medline, Latindex, Google Scholar data bases were searched to retrieve reports from 1951 up to 2012 regarding prevalence data, diagnostic tests and risk factors of infection among the adult population. Data collection and criteria eligibility was established in order to determine the crude prevalence (proportion of positive cases) of each study, together with weighted population prevalence according to individual research group categories to limit the bias that may impose the heterogeneous nature of the reports. A Forest Plot chart and linear regression analysis were performed by plotting the prevalence of infection reported from each study over a period of sixty years.

**Results:**

A total of 132 studies were collected from 41 publications that included 70,123 individuals. The average mean prevalence was 27.97%, and weighted mean prevalence was 19.27%. Comparisons among different risk groups showed that the weighted prevalence was higher in women with miscarriages (36.03%), immunocompromised patients (28.54%), mentally-ill patients (38.52%) and other risk groups (35.13%). *Toxoplasma* infection among the Mexican population showed a downward trend of 0.1%/year over a period of sixty years that represents a 5.8% reduction in prevalence.

**Conclusions:**

This analysis showed a downward trend of infection; however, there are individuals at high risk for infection such as immunocompromised patients, mentally-ill patients and pregnant women. Further research is required to provide better prevention strategies, effective diagnostic testing and medical management of patients. Educational efforts are required to avoid the transmission of infection in populations that cannot be controlled by drugs alone.

## Background

Toxoplasmosis is a disease caused by *Toxoplasma gondii (T. gondii)*. It was described in a North African rodent (*Ctenodactylus gondii*) by Nicolle and Manceaux in 1908 [1]. The *T. gondii* is an obligate intracellular parasite with a complex life cycle, in which homeothermic animals, including humans are capable of acting as intermediate hosts. Humans acquire the parasite by the oral route through the consumption of undercooked meat contaminated with cysts, food products (vegetables and fruits) or water contaminated with oocysts [1,2]. Other routes of transmission are organ transplantation [3,4], blood transfusion [5] and congenital transmission. Butchers, slaughterhouse workers and laboratory personnel that handle cultures and animal models with this parasite are also at risk. However, for the majority of the human population, transmission generally occurs by any of the routes aforementioned [6].

*T. gondii* is found worldwide because a large variety of animals may harbor the parasite and maintain its dissemination. Its broad geographic location is related to several factors, such as contact with infected cat feces and ingestion of mature oocysts [7], food habits and variations in climate. The later has a significant influence on the habitat of *T. gondii;* for instance, an increase in ambient temperature and precipitation can change the humidity of the soil, so that the sporulated oocysts remain viable in the moist environment for a longer period [8,9].

*T. gondii* is considered as the most prevalent parasitic zoonotic disease worldwide [10], since at least one-third of the world’s population is infected [11]. Infections caused by *T. gondii* are more frequent in temperate zones than in cold ones; thus, France has the highest prevalence of 90%, whereas the lowest prevalence is found in Alaska with only 1%. However, global warming has caused an increase of *T. gondii* infections in different regions of the world as a result of changing environmental conditions [8].

The mean prevalence of *T. gondii* infection among the Mexican general population is 50%; however, there are variations that depend on climate and humidity. Several risk groups have been identified with high prevalence of infection such as cat owners, people who consume raw or undercooked meat, immunocompromised patients and those that undergo organ transplantation [3,4]. Furthermore, we recently carried out a meta-analysis on reports of toxoplasmosis among Mexican newborns. The weighted prevalence in 4833 asymptomatic newborns was 0.616%, whereas, among 895 symptomatic newborns, the weighed prevalence was 3.02% [12].

Diagnostic testing for toxoplasmosis can be done by staining body fluids or mouse inoculation to see if Toxoplasma parasites develop. Skin test antigen (toxoplasmin) (STA), and serological procedures such as the Complement Fixation Test (CF), Latex Flocculation Test (LF), Sabin and Feldman (SF), Indirect Haemagglutination Test (IHA), Indirect Immunofluorescence assay (IFI), and Enzyme-linked immunosorbet assay (ELISA) have been employed to detect specific antibodies in screening programs and also as adjuncts to the diagnosis of acute toxoplasmosis. More recently other methods have been developed such as Western-blot and detection of DNA with polymerase chain reaction (PCR) [1,4].

Treatment for human toxoplasmosis is highly important for immunocompromised patients or acutely infected pregnant women. Various pharmacological agents are available such as pyrimethamine alone or combined with sulfadiazine. Atovaquone has been used as a second course of treatment for retinochoroiditis. Azithromycin is used as an alternative in the treatment of ocular and cerebral toxoplasmosis in AIDS patients, as well as, for active, non-vision-threatening toxoplasmic retinochoroiditis with satisfactory results [1,13,14].

The purpose of this systematic review and meta-analysis was to evaluate the seroprevalence of toxoplasmosis and its relationship with different risk factors. The understanding of these relationships can aid in the analysis of the epidemiological pattern of disease among different population groups and the epidemiologic shift of *T gondii* infection in Mexico.

## Methods

### Ethical Aspects

This study was approved by the Ethical Committee of the Health Sciences Center of the University of Guadalajara # C.I.100-2012.

### Database search

Five databases were searched (Pub Med, Lilacs, Medline, Latindex and Google Scholar) from January to June of 2012. The following limits were applied: published January 1951 to 2012, the first case of human *T. gondii* infection was reported in an 11-month old girl from the Pediatric Hospital at Mexico City in 1950 [15], written in English or Spanish and undertaken in adults. The search terms were “infection with *Toxoplasma”,* “toxoplasmosis”, “epidemiology”, “risk factors”, “infection by *T. gondii*, Mexico” “anti-*Toxoplasma* antibodies” alone or combined.

### Data collection

All retrieved studies were studied carefully by two investigators (GRML and BR). The extracted data included: year of publication, characteristics of the study population, location of the study, sample size, number of cases, diagnostic test and risk factors. Abstracts were included if considered acceptable, but were not included in the meta-analysis for risk factors. Reference lists of full-text publications and textbooks were also examined to identify studies not retrieved by the original search.

### Data analysis

The crude prevalence data and the weighted prevalence were calculated for each study (Table 1) [16-59]. Seven different types of diagnostic tests were identified during the literature search. Table 2 summarizes their main characteristics, sensitivity and specificity, and their timeline of employment in Mexico. Study groups were also stratified according to categories alone (Table 3) or by combining the risk group and diagnostic test used in each study [60-66] (Table 4).

**Table 1:** Publications included for meta-analysis with diagnostic methods and population characteristics

**Sequence**	**Year**	**First Author**	**State**	**Municipality**	**Test**	**Category**	**Number of cases**	**Positive cases**	**Prevalence (%)**	**Reference**
1	1951	BiagiF.	Tamaulipas	Tampico	STA	GP	231	108	47	[16]
2	1952	Biagi F.	México	Mexico	STA	IC	155	58	37.4	[49]
3	1952	Bustos C.	Veracruz	Orizaba	STA	GP	86	31	51	[17]
4	1953	Biagi F.	Campeche	Escarcega	STA	GP	132	76	56.8	[18]
5	1953	Varela G.	Estado de Mexico	Toluca	STA	GP	500	81	16	[19]
6		Varela G.	Mexico	México	STA	CM	116	19	16	[19]
7		Varela G.	Mexico	México	STA	CM	47	7	14.8	[19]
8		Varela G.	Mexico	México	STA	CM	102	13	12.7	[19]
9		Varela G.		Zoquiapan	STA	IC	107	13	14	[19]
10	1954	Gutierrez E.B.	México	México	CFT	MI	58	14	24.14	[53]
11	1955	Varela G.	Mexico	México	SF	GP	60	16	26.7	[20]
12		Varela G.	Michoacan	N.E.	SF	GP	22	6	27.3	[20]
13		Varela G.	Mexico	México	SF	GP	104	39	37.5	[20]
14		Varela G.	Oaxaca	Oaxaca	SF	GP	276	96	34.8	[20]
15		Varela G.	Tamaulipas	N.E.	SF	GP	230	90	39	[20]
16		Varela G.	Yucatan	N.E.	SF	GP	17	11	64.7	[20]
17		Varela G.	Distrito federal	Mexico	SF	MI	91	54	59	[20]
18		Varela G.	Puebla	N.E.	SF	GP	44	41	93.2	[20]
19	1957	Biagi F.	México	Ixtapalapa, D.F.	STA	GP	272	37	13.6	[21]
20	1961	Varela G.	Baja, California	Mexicali	SF	GP	73	26	35.9	[22]
21		Varela G.	Chihuahua	Chihuahua	SF	GP	12	3	25	[22]
22		Varela G.	México	Distrito Federal	SF	GP	2,463	783	31.8	[22]
23		Varela G.	Hidalgo	Apan	SF	GP	409	117	28.6	[22]
24		Varela G.	Estado de Mexico	Toluca	SF	GP	64	15	23.4	[22]
25		Varela G.	Michoacan	Morelia	SF	GP	35	11	31.42	[22]
26		Varela G.	Morelos	Various	SF	GP	208	54	25.96	[22]
27		Varela G.	Nayarit	Tepic	SF	GP	112	25	22.3	[22]
28		Varela G.	Oaxaca	Oaxaca and Tuxtepec	SF	GP	546	107	19.59	[22]
29		Varela G.	Puebla	Puebla	SF	GP	170	61	35.9	[22]
30		Varela G.	Queretaro	Various	SF	GP	90	33	36.66	[22]
31		Varela G.	Sinaloa	Culiacan	SF	GP	311	109	35	[22]
32		Varela G.	Sinaloa	Mazatlan	SF	GP	100	16	16	[22]
33		Varela G.	Tabasco	Macuspana	SF	GP	108	38	35.2	[22]
34		Varela G.	Tamaulipas	Ciudad Victoria	SF	GP	220	39	17.7	[22]
35		Varela G.	Tamaulipas	Nuevo Laredo	SF	GP	100	28	28	[22]
36		Varela G.	Tlaxcala	Tlaxcala	SF	GP	594	144	24.2	[22]
37		Varela G.	Veracruz	Boca del Rio	SF	GP	93	36	38.7	[22]
38		Varela G.	Veracruz	Soconusco	SF	GP	121	39	32.2	[22]
39		Varela G.	Veracruz	Veracruz	SF	GP	125	30	24	[22]
40		Varela G.	Yucatan	Merida	SF	GP	17	11	64.7	[22]
41	1962	Carrillo C.	México	Mexico	SF	BD	232	73	31.4	[46]
42	1965	Espinosa de los Reyes VM,	México	Mexico	SF	PW	329	112	34	[37]
43	1966	Roch E.	Distrito Federal	México	SF	MW	2,320	815	35.13	[44]
44	1966	Roch E.	Mexico	All States	SF	GP	14,869	4,411	30	[23]
45	1972	Goldsmith RS.	Oaxaca	Puerto Escondido	IHA	GP	159	2	1.26	[24]
46	1972	Goldsmith RS.	Oaxaca	Mixteca Alta	IHA	GP	114	0	0	[24]
47	1972	Goldsmith RS.	Oaxaca	Ixtlan	IHA	GP	48	2	4.2	[24]
48	1972	Goldsmith RS.	Oaxaca	Región del Valle	IHA	GP	150	5	3.3	[24]
49	1972	Goldsmith RS.	Oaxaca	Tehuantepec	IHA	GP	137	18	13	[24]
50	1974	Biagi F.	Mexico	México	FL	WP	367	73	19.9	[35]
51	1986	Fernandez Terrano	Tabasco	Region de los Rios	IFI-G	WP	125	75	60	[36]
52	1989	Galvan-Ramirez ML.		Jalisco	IFI-G	GP	807	25	3.1	[25]
53	1989	Zavala-Velazquez J.	Yucatan	Merida	IFI-G	MW	100	47	47	[45]
54	1991	Goldsmith RS.	Oaxaca	60 municipalities	IHA	GP	3,229	124	3.8	[26]
55-86	1991	Velasco- Castrejon O.	Mexico	All states	IFI-G	GP	29,279	9,371	32	[27]
87	1995	Galvan-Ramirez ML.	Jalisco	Guadalajara	ELISA-G	PW	350	122	34.9	[39]
88		Galvan-Ramirez ML	Jalisco	Guadalajara	ELISA-G	MW	105	48	44.9	[39]
89		Galvan-Ramirez ML	Jalisco	Guadalajara	ELISA-G	PW	50	13	26.01	[39]
90	1997	Galvan-Ramirez ML	Jalisco	Guadalajara	ELISA-G	IC	39	27	69.2	[50]
91		Galvan-Ramirez ML	Jalisco	Guadalajara	ELISA-G	IC	53	19	35.8	[50]
92	1997	Tay J.	Distrito Fed.	México	ELISA-G	MI	328	125	38	[54]
93	1998	Gongora R.	Yucatan	Mérida	ELISA-G	IC	95	45	47	[47]
95		Gongora R.	Yucatan	Mérida	ELISA-G	BD	100	69	69	[47]
93	1999	Galvan-Ramirez ML	Jalisco	Guadalajara	ELISA-G	ORG	59	38	64	[7]
96	2000	Kelso Santos E.	Nuevo Leon	Monterrey	ELISA-G	GP	400	82	20.5	[29]
97	2003	Jaramillo P.J.	Estado de Mexico	Toluca	ELISA-G	PW	372	47	12.61	[40]
98	2004	Galvan-Ramirez ML	Jalisco	Guadalajara	ELISA-G	PW	30	14	47	[38]
99		Galvan-Ramirez ML	Jalisco	Guadalajara	ELISA-G	PW	30	13	43	[38]
100		Galvan-Ramirez ML	Jalisco	Guadalajara	ELISA-G	PW	60	17	28.3	[38]
101	2005	Galvan-Ramirez ML	Jalisco	Guadalajara	ELISA-G	BD	359	104	29	[5]
102	2006	Alvarado-Esquivel C.	Durago	Guadalajara	ELISA-G	PW	343	21	6.1	[41]
103	2007	Alvarado-Esquivel C.	Durango	Durango	ELISA-G	BD	432	32	7.4	[48]
104	2008	Alvarado-Esquivel C.	Durango	Durango	ELISA-G	ORG	90	19	21.1	[58]
105		Alvarado-Esquivel C.	Durango	Durango	ELISA-G	ORG	83	7	8.4	[58]
106	2008	Alvarado-Esquivel C.	Durango	Durango	ELISA-G	GP	187	67	35.8	[30]
107	2008	Alvarado-Esquivel C.	Durango	Durango	ELISA-G	GP	121	20	16.5	[30]
108	2008	Alvarado-Esquivel C.	Durango	Durango	ELISA-G	GP	155	23	14.8	[30]
109	2008	Galvan-Ramirez ML.	Jalisco	Guadalajara	ELISA-G	ORG	145	104	72	[6]
110	2009	Alvarado-Esquivel C.	Durango	Guadalajara	ELISA-G	PG	439	36	8.2	[42]
111	2009	Cañedo-Solares I.	Distrito Federal	Mexico	ELISA-G	PG	100	30	30	[43]
112	2010	Alvarado-Esquivel C.	Durango	Durango	ELISA-G	GP	248	22	8	[31]
113	2010	Alvarado-Esquivel C.	Durango	Durango	ELISA-G	GP	61	4	6.6	[56]
114		Alvarado-Esquivel C.	Durango	Durango	ELISA-G	GP	203	17	8.4	[56]
115		Alvarado-Esquivel C.	Durango	Durango	ELISA-G	GP	168	10	6.6	[56]
116	2010	Alvarado-Esquivel C.	Durago	Durango	ELISA-G	GP	152	46	30	[31]
117	2010	Galvan-Ramirez ML	Jalisco	Guadalajara	ELISA-G	GP	174	30	17.8	[28]
118	2010	Alvarado-Esquivel C.	Durango	Durango	ELISA-G	CM	85	7	8.2	[51]
119		Alvarado-Esquivel C.	Durango	Durango	ELISA-G	CM	50	5	10	[51]
120		Alvarado-Esquivel C.	Durango	Durango	ELISA-G	CM	234	28	12	[51]
121		Alvarado-Esquivel C.	Durango	Durango	ELISA-G	IC	103	7	6.8	[51]
122	2011	Alvarado-Esquivel C.	Durango	Durango	ELISA-G	ORG	124	8	7	[57]
123	2011	Alvarado-Esquivel C.	Durango	Durango	ELISA-G	MI	50	10	20	[55]
124	2011	Alvarado-Esquivel C.	Durango	Durango	ELISA-G	GP	150	8	5.3	[32]
125	2011	Alvarado-Esquivel C.	Durango	Durango	ELISA-G	IC	75	10	13.3	[52]
126		Alvarado-Esquivel C.	Durango	Durango	ELISA-G	GP	150	16	10.7	[52]
127	2011	Alvarado-Esquivel C.	Durango	Durango	ELISA-G	GP	1,101	76	6.9	[59]
128		Alvarado-Esquivel C.	Durango	Durango	ELISA-G	CM	55	9	16.4	[59]
129	2011	Alvarado-Esquivel C.	Durango	Durango	ELISA-G	GP	974	59	6.1	[32]
130	2012	Alvarado-Esquivel C.	Durango	Durango	ELISA-G	GP	133	11	8.3	[33]
131		Alvarado-Esquivel C.	Durango	Durango	ELISA-G	GP	266	14	5.3	[33]
132	2012	Alvarado-Esquivel C.	Durango	Durango	ELISA-G	GP	156	35	22.4	[34]
**Total**							**70,123**	**19,262**		

Skin test antigen (toxoplasmin) (STA); Complement Fixation Test (CF); Latex Flocculation Test (LF); Sabin & Feldman (SF); Indirect Haemagglutination Test (IHA); Enzyme-linked immunosorbet assay and type of antibody detected (ELISA-G), Indirect Immunofluorescence assay and type of antibody detected (IFI-G); General Population (GP); Pregnant Women (PW); Blood Donors (BD); Patients with Comorbidity (CM); Immunocompromised patients (IC); Women with Miscarriages (MW); Mentally-ill patients (MI); Other risk groups (ORG).

**Table 2:** Diagnostic methods

**Diagnostic test**	**Fundament**	**Sensitivity**	**Specificity**	**Timeline**	**References**
Skin test antigen (STA	Type IV cell-mediated hypersensitivity reaction against the *T.gondii* antigen.	80%	70%	1950-1951	[16-19,21,49]
Sabin and Feldman Dye Test (SF).	The gold standard. A dye test in which the serum antibodies alter the staining pattern of the *T. gondii* tachyzoites.	96%	98%	1955-2005	[20,22,23,37,44,46]
Complement Fixation Test (CF).	Antigen-antibody complexes are formed and detected by using a standard system with hemolisin and complement	97.1%	64.5%	1954-1982	[53]
Látex Flocculation Test (LF).	This test uses latex particles for antigen-antibody flocculation.	No reported.		1974	[35]
Indirect Fluorescent Antibody Test (IFI).	Tachyzoites are fixed on a slide and exposed to test serum, then washed and exposed to a standard antibody labeled with fluorescent dye.	95%	96%	1986 to date	[5,25,27,28,36,38,54]
Indirect Haemagglutination Test (IHA).	This test uses sheep red cells exposed to tannic acid and then to the soluble antigen fixed at 37 °C.	95%	96%	1972 to 1989	[24,26]
Enzyme-linked immunoabsorbent assay (ELISA).	The ELISA detects *T. gondii *immunoglobulin IgG e IgM in serum and other body fluids with antibodies marked with peroxidase and fosfatase enzymes	100%	98.4%	1995 to date	[6,7,29-34,39-43,47,48,50-52,55,56]

**Table 3:** **Crude and weighted *Toxoplasma *** **infection prevalence in low and high risk groups of Mexican population**

**Population studied**	**Number of studies**	**Number of cases**	**Positive cases**	**A/Nx100 Crude prevalence (%)**	**Weighed prevalence (%)**	**CI 95% Lower-upper limit (%)**	**References**
**Low Risk Groups**							
General population	90	61536	16855	27.39	20.26	18.78-19.36	[16-24,34,51,52,55,56,58,59]
Pregnant Women	12	2595	573	22.08	15.62	14.30-16.93	[35-43]
Blood Donors	4	1123	278	24.76	17.035	15.03-19.03	[5,46-48]
Patients with comorbidity	7	689	88	12.77	12.27	9.83-14.72	[19,51,59]
**High Risk Groups**							
Women with miscarriages	3	2525	910	36.03	35.96	34.1-37.83	[39,44,45]
Immunocompromised patients	7	627	179	28.54	20.2	17.35-23.05	[19,47,49-52]
Mentally-ill patients	4	527	203	38.52	37.24	33.24-41.26	[20,53-55]
Other risk groups	5	501	176	35.13	21.88	19.0-24.76	[6,7,57,58]
**Total**	**132**	**70,123**	**19,262**				

**Table 4:** **Weighted prevalence of** ***T. gondii*** **infection adjusted by risk factor (with or without) and diagnostic method**

**Population studied-diagnostic method**	**Number of groups**	**Number of cases**	**Positive cases**	**A/Nx100 prevalence (%)**	**Meta-analysis prevalence (%)**	**Lower limit (%)**	**Upper limit (%)**
with Risk - STA	4	480	103	21.45	13.39	10.35	16.44
without Risk -STA	6	1268	340	22.81	22.48	20.32	24.65
without Risk-LF	1	367	73	19.89	19.89	15.8	23.97
without-risk CF	1	58	14	24.14	22.16	13.64	35.15
without Risk-SF	32	22245	6674	30	30	29.4	30.59
with Risk-SF	1	2320	815	35.13	35.12	33.18	37.18
without Risk-IFI-G	36	29997	9597	31.99	29.3	28.81	29.79
with Risk-IFI-G	5	1295	224	16.77	5.58	4.44	6.77
without Risk-IHA	6	3837	151	3.94	2.63	1.24	4.02
with Risk-ELISA-G	18	1963	523	26.64	17.66	16.17	19.15
without Risk-ELISA-G	22	6293	748	11.89	8.92	8.24	9.6
**Total**	**132**	**70,123**	**19,262**				

Complement Fixation (CF), Skin test antigen toxoplasmin (STA), Latex Floculation (LF), Sabin and Feldman (SF), Indirect Hemaglutination (IHA), Indirect Fluorescence (IFI), Enzyme-linked immunosorbet assay (ELISA). All prevalences calculated by meta-analysis were statistically significant (p < 0.001).

The study groups were divided into: 1) Individuals with risk factors were designated as high risk groups that included: women who had had abortions, immunocompromised patients with AIDS or HIV, leprous people, patients with neurological disorders, pet-cat, owners and slaughterhouse workers; 2) Individuals without risks factors were designated in low risk groups: blood donors, general population and normal pregnant women.

### Statistical methods

#### ***Crude prevalence***

The crude prevalence of each study group was estimated to assess the amount of affection by the disease expressed in percentage of positives cases in relation to all cases analyzed. It was calculated as the number of positive cases divided by the sample size of the cohort in each study group.

#### ***Weighted population prevalence (WP)***

The assessment of the prevalence of *T. gondii* of the different study groups was estimated by using the weighed population prevalence, given that not all the studies included the same number of individuals. This strategy restricts the bias that may impose the heterogeneous nature of the reports, and has proven to be valid when combining a number of studies with inherent heterogeneity in sample size and effects [67].

Each crude prevalence was multiplied by a “weight”, which was proportional to the number of subjects included in the sample, i.e., in large samples, the prevalence found outweighs that of small samples. This prevalence is obtained by summing the product of the prevalence for its “weight” of the sum of the “weights”. This estimate is more accurate than the overall crude prevalence to estimate the true prevalence of a cumulative set of groups. The formula to calculate the population prevalence (weighed prevalence, WP) of *T. gondii* in all groups or subgroups, included in this meta-analysis was P = ∑ (pi)(1/vi)/∑ 1/vi as explained by Borenstein et al. [68].

### Definitions

i = Number of studies in each group. Ni = Total number of cases in each study, Ai = Number of positive cases from each study, (Ni-Ai) is the number of negative cases in each study. The risk of infection as a proportion in each study (pi) was calculated as Ai/Ni. The variance of each study (vi) was calculated as Ai (Ni-Ai)/Ni^3^. The standard error (SEi) of each study was estimated as √ vi.

The total population variance (V) was estimated as 1/ ∑ 1/vi. The standard error of the population was calculated as SE = √V. The confidence interval (C.I.95%) for the population prevalence was obtained by P + 1.96 SE (upper limit) and P-1.96 EE lower limit. The probability that the prevalence could be different from zero was calculated with a Z test, Z = P/SE.

#### ***Forest plot***

A Forest Plot chart was built in order to provide a comprehensive analysis of the studies included in the meta-analysis according to its odd ratio and confidence interval (CI) [68].

#### ***Linear regression analysis***

A bivariate linear regression analysis was conducted to determine the relationship between the seroprevalence of *Toxoplasma* infection over time. The regression coefficient was calculated by the equation (y = a + b x), a = ordinate of origin, b = slope and the R^2^ and p were obtained with the SPSS program (Version 18). The epidemiological behaviour of the prevalence of *Toxoplasma* infection was estimated by plotting the year of each publication date (independent variable) starting at year 1951 until 2012 versus the relative prevalence (dependent variable) reported in each study [68].

## Results

From the five databases, a total of 45 publications were eligible that included 132 studies and 70,123 individuals, and 19,262 positive cases as shown in Table 1. As shown in Table 3, most of the studies were carried out in low risk groups, such as, the general population (n = 90 studies) followed by pregnant women (n = 12), with the least number of studies in blood donors (n = 4). In the high-risk groups, relatively fewer studies have been carried out; women with miscarriage (n = 3), immuno-compromised patients (n = 7), mentally-ill patients (n = 4), other risk groups (n = 5) and patients with nonrelated comorbidity (n = 7). To the best of our knowledge, all the studies included in this meta-analysis had a cross-sectional design and were aimed to identify the prevalence of *T. gondii* antibodies in a cohort. No prospective or follow-up studies aimed to seek seroconversion or self-reported results were detected.

### Meta-analysis in low risk groups

#### ***General population***

Most of the studies were carried out in low risk groups such as the general population (90 studies) that gave a total of 61,536 people tested for *T. gondii infection*. The WP was 20.26% (CI_95%_18.78% – 19.36%) with a variance of 0.0002% and a standard error of 0.1463%, Z = 130.391 and p <0.001 (Table 3).

#### ***Pregnant women***

In this study group, 12 publications included 2,595 pregnant women. The WP was 15.62% (CI_95%_14.30%-16.93%), with a variance of 0.0045% and standard error of 0.67%, Z = 23.313 and p <0.001 (Table 3).

#### ***Blood donors***

In 4 studies, a total of 1123 blood donors were tested. The WP was 17.03% (CI_95%_ 15.03% – 19.03%) with a variance of 0.00104% and standard error of 1.0213%, Z = 16.679 and p <0.001 (Table 3).

#### ***Patients with comorbidity***

In 689 cases from seven studies, a WP of 12.27% (CI_95%_ 9.83 – 14.72) was found with a variance of 0.0155%, standard error of 1.2463%, Z = 9.85 and p <0.001 (Table 3).

### Meta-analysis in high-risk groups

#### ***Women with miscarriages***

In 3 studies, 2595 women were tested. The WP was 35.96% (CI_95%_ 34.10% - 37.83%) with a variance of 0.0091% and standard error of 0.9532%, Z = 37.73 and p <0.001 (Table 3).

#### ***Immunocompromised patients***

In this study group, 627 patients from seven studies were included. The WP was 20.20% (CI_95%_ 17.35%- 23.05%) with a variance of 0.0211% and standard error of 1.45%, Z = 13.90 and p <0.0001 (Table 3).

#### ***Mentally-ill patients***

A total of 527 mentally-ill patients were included from four studies. The WP was 37.24% (CI_95%_ 33.24% - 41.26%) with a variance of 0.0994% and standard error of 3.15%, Z = 11.43 and p <0.001 (Table 3).

#### ***Other risk groups***

In 501 cases from five studies, a WP of 21.88% (CI_95%_ 19.00% - 24.76%) was found with a variance of 0.0215%, standard error of 1.46%, Z = 14.91 and p <0.001 (Table 3).

#### ***Weighted prevalence by diagnostic test and risk factors***

In order to determine, if the prevalence of *T. gondii* was based on the diagnostic test, the study groups were adjusted by diagnostic test and risk factors reported. Only the studies that used the SF assay and ELlSA showed a correlation between the low and high risk. Interestingly, the rest of the diagnostic tests showed a lower prevalence, although it is noteworthy to mention that the difference may be due to the number of cases in the risk group categories (Table 4).

#### ***Epidemiological transition of Toxoplasma infection in Mexico***

**Figure 1:**
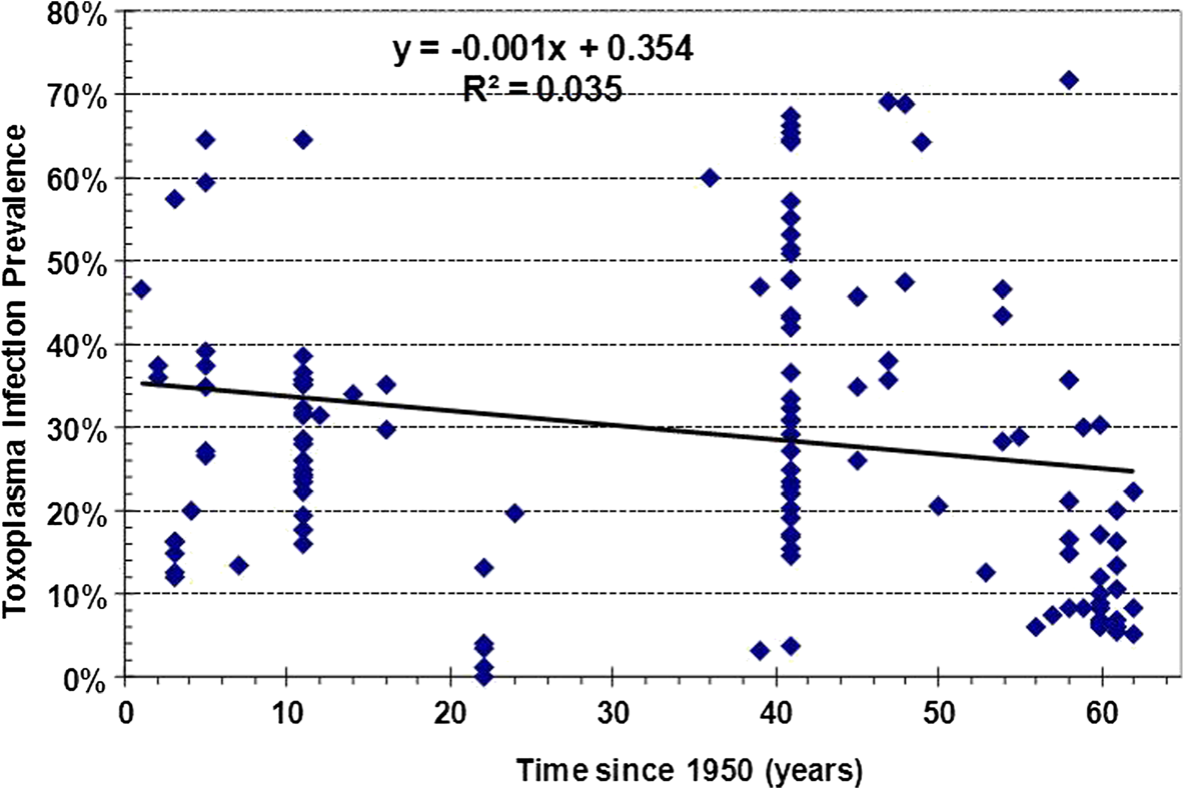
**Epidemiological transition of the *T. gondii *infection from 1954 to 2012, (―) Linear regression and triangle (♦) is the individual prevalence of *Toxoplasma gondii *infection reported in each study group. **The decrement in the prevalence rate was 0.1%/year. The R^2 ^value was not statistically significant (NS).

**Figure 2:**
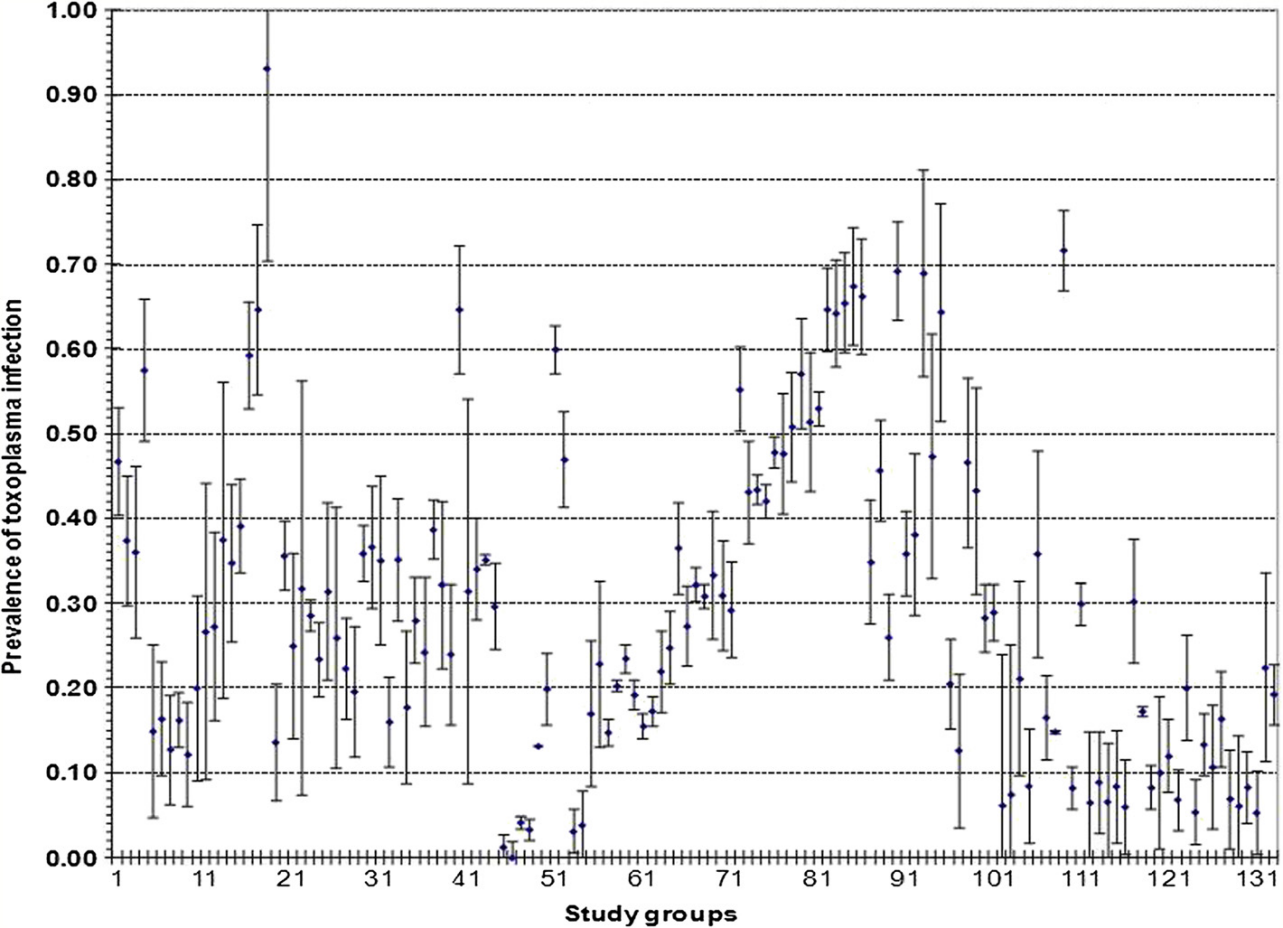
**Weighted prevalence of *Toxoplasma gondii *infection from 132 studies. **Forest plot shows event numbers, total numbers and confidence intervals for all study groups included in the meta-analysis. The triangle (♦) represents the prevalence of each study and (┬ ┴) are intervals of confidence.

Figure 1 illustrates the Forest Plot analysis of the WP (CI_95%_) of each study included in this meta-analysis. The linear regression analysis of *Toxoplasma* infection in the Mexican population over a time span of 60 years showed a downward trend of 0.1%/year that represents an overall 5.8% reduction of infection (R^2^ = 0.0354 and F (1) =4.802, p > 0.05) that was not statistically significant (Figure 2).

## Discussion

The questions that led us to achieve this study were if Mexico had a significant prevalence of toxoplasmosis, and if there have been changes in the epidemiology of this disease from 1951 to date. Five databases were searched, and 132 studies were selected involving 70,123 individuals and 19,262 positive cases.

This study showed that the WP of toxoplasmosis in Mexico was 20.26%, which is relatively low compared to the mean prevalence of 32% reported in 1991 [27]. This can be explained if we note that variations between methodologies can result in heterogeneity of the estimations. However, despite this situation, at least 20.26% of Mexicans may have been exposed to toxoplasmosis.

Between 1951 and 1958, the STA was an appropriate test since, at that time, it was a rapid and sensitive assay for epidemiological studies. It did not give cross-reactions with other parasites, but it did make a fair amount of false-negatives in mild infections. This was one of its disadvantages when used for a survey in the general population in Tamaulipas, Campeche and Mexico City (1953). The WP was 21.31% in 11 study groups with 1846 cases [16-20]. Since only one study was performed by the FL test with 367 individuals, and another study included 58 cases tested by fixation complement, we were not able to compare these results against the SF or IFI assays.

The SF test was used in epidemiological studies carried out between 1961 and 1966. The CP and WP was 30% and 35.12%, respectively [23]. The advantage of this test is that it is a gold standard, and it is highly sensitive, although the disadvantage is that it requires live parasites. The global prevalence during that time was 14.92%, which is closer, to the prevalence of 8.26% aforementioned. In the same period, the IFI test was introduced and was then used in the largest survey carried out in Mexico (1992) with 29, 279 people with a mean prevalence of 32.0% [27]. The meta-analysis of all tests performed with IFI was 29.32%, because the majority of studies have used this method (31,412 cases).

In the 80s, the ELISA methods were introduced with higher sensitivity and specificity that reduced the number of cross-reactions. Interestingly, the WP was lower, ranging from 8.92% to 17.66%, which may have been caused by the lower number of cases, (8256 cases), less than one-third of the studies, when compared to the 26,751 cases tested with SF and the 30,485 cases with IFI.

The general population involved 61,536 cases with a WP of 20.26%, while the mean prevalence was reported as 32% in 1991 [27]. This discrepancy may be caused by the differences in the diagnostic methods that are then adjusted when the weighted prevalence was estimated [27].

Pregnant women presented a WP of 15.62% with an upper limit of 16.93% that is closer to the general population prevalence (20.26%), which is reasonable since these women did not have obstetric complications. These values were lower compared to 45.8% and 30.5%, respectively for specific IgG antibodies detected by the Sabin-Feldman dye test in two separate studies among pregnant women [69], however, our results were higher than 0.6% among pregnant women in Norway [70].

In the four studies performed in blood donors, the WP was 17.03% among 1123 cases. This prevalence is also closer to the general population prevalence, despite that each one of them was carried out with different diagnostic tests (one with SF [46], one not reported [47], one with IFAT [5] and one with ELISA, respectively [48]).

Regarding the blood donors group, this group has a lower WP when compared to the general population. Transfusion Medicine guidelines have pronounced that donated blood should be screened for toxoplasmosis, because of the potential risk for blood receptors receiving transfusions from subjects in the acute phase of infection. Therefore, testing of toxoplasmosis in blood donations should be mandatory in the country [5,46-48].

Three studies performed in Mexico were in women with miscarriages having primary infection during pregnancy. There were clear differences between the WP of pregnant women 15.62% whereas those with miscarriages had 35.96% (p <0.05). These results are concordant with other studies that have shown an association between high prevalence of miscarriages and *Toxoplasma* infection [39,44,45].

Another risk group was the immunocompromised patients. Among 627 cases, the WP of 20.2% was the same as in the general population. One explanation is that the patients were diagnosed by different methods, and two studies performed in Durango had the lowest prevalence [47,49-52].

The highest WP was 37.24% in the mentally-ill patients, at least 18.27% higher than the general population [55]. Several authors have reported a high prevalence of *Toxoplasma* antibodies in patients with schizophrenia, although other factors such as genetic may be present in the schizophrenia, different reports have shown that Toxoplasma is somehow associated with cases of schizophrenia. This finding justifies the need to examine the relationship between toxoplasmosis and schizophrenia with pre-clinical and clinical trials aimed to improve prevention and treatment programs in patients with psychiatric illness [71,72]. On the other hand, a meta-analysis of latent *Toxoplasma gondii* infection in immunocompetent hosts and cryptogenic epilepsy showed a strong association between seroprevalence rates for toxoplasmosis and prevalence rates of epilepsy. If an etiological connection can be proven, it would have implications for the implementation of prevention and treatment strategies for Toxoplasma disease [73].

Cat owners and slaughterhouse workers presented both a CP and WP of 21.88% each. This may be because only five studies with 501 individuals with this attribute were analyzed. Regarding patients with nonrelated comorbidity, WP was 12.28%, lower than in the general population. Three groups were studied with STA and four more from Durango had the lowest prevalence in the country, which may show that their comorbidity was not related to the risk of infection.

Another important factor is the difference in the prevalence of *T. gondii* infections due to the sensitivity and specificity of diagnostic tests, since there are several methods to identify and evaluate antibodies in individuals who were infected by the parasite. Over time, at least four different diagnostic assays have been used worldwide that range from the lowest specificity and sensitivity like the STA progressing on to the SF Dye Test and other similar tests up to the improved ELISA (Table 2) [7,74].

In regards to the analysis of the study groups adjusted by risk factors (with or without) and diagnostic test, we found that only those tested by ELISA showed a WP of 17.66% in the risk factor groups against a WP of 8.92% in the groups without risk factors. However, with the other methods, both crude and weighted prevalence were reduced; however, the number of studied individuals was higher. This analysis demonstrates that the prevalence varies according to the diagnostic method and by the number of individuals tested in each study group (Figure 2).

The epidemiological behavior of *Toxoplasma* infection showed a negative slope of -0.1%/year, which represented an accumulated decrement of nearly 6% in the prevalence of infection in Mexico after 60 years. This may suggest that we have not paid enough attention to *T. gondii* infection as a public health problem though it tends to decrease. However, another key issue is that if the studies carried out in the first 10 years had been tested with ELISA then the decrement would have been even lower (Figure 2).

In this study, stratification of studies based on molecular assays was not feasible because none was reported. However, genotyping has been reported in other Latin American populations, such as Colombia where a virulent strain (LD_100_ of 10 tachyzoites) was identified as clonal type 1 (CIBMUQ/HDC) [75]. Additionally, in another study, the GRA6 type I/III profile was the most frequent among asymptomatic cases (68/148, 45.9%) and in severe multi visceral cases (2/4, 50%). Furthermore, GRA6 type II, was found in one case of congenital toxoplasmosis, one case of severe multi visceral infection, one case of ocular infection, and in five cases (5/148,3.4%) of asymptomatic patients [76]. Further studies based on genetic-based diagnostic assays will be relevant in the future, since *Toxoplasma gondii* isolates from Latin America have mixed/ recombinant genetic structure.

A 6% decrement in the prevalence of *Toxoplasma* infection after 60 years of studies was detected. This decrement is quite low after a relatively long period, contrary to what has been reported in the United States, with a 14% decrement in only one decade [77]. Therefore, our data warrants that researchers must pay more attention to this disease and to communicate to the medical community the need of improvement of the prevention strategies, together with effective diagnostic testing and management of patients in high risk of infection such as immunocompromised patients and pregnant women.

Further research is still required to understand and clarify the role of *T*. *gondii* in its diverse routes of transmission, as well as to design better control measures that focus on minimizing the risk of infection. The purpose of such studies should be to aid in the monitoring of changes in the epidemiology of *T. gondii* infection, and to strengthen educational efforts in order to avoid the transmission of infection in populations that cannot be controlled by drugs alone.

### Implications for research

A crucial factor is the difference in the prevalence of *T. gondii* infections due to the sensitivity and specificity of the diagnostic tests, since there are several methods to identify and evaluate antibodies in individuals who were infected by the parasite. At least four different diagnostic assays have been used in this study, that range from the lowest specificity and sensitivity like the CF, progressing on to the SF dye test and other similar tests up to the improved ELISA.

## Conclusion

In conclusion, to the best of our knowledge, this is the first study that provides a comprehensive view of the epidemiological situation on the prevalence of *T. gondii* infection among the adult Mexican population. It provides not only epidemiologic evidence relevant to Mexico, but to other countries in the Americas and worldwide as well, where it has been documented that *T. gondii* prevalence is shifting, related to regional climate changes among other factors. In this study, the major risk groups with Toxoplasma infection were women with miscarriages, immunocompromised patients, mentally-ill patients and other risk groups. Noteworthy was the psychiatric patients group, since *T.gondii* can cause serious damage to the central nervous system.

## Competing interests

The authors declare that they have no competing interests.

## Authors’ contributions

MLG conceived and designed the research, RT performed statistical tests, MLG and SR wrote the manuscript, CC and RB database information search. All authors read and approved the final version of the manuscript.
